# Identification of SNPs in *MITF* associated with beak color of duck

**DOI:** 10.3389/fgene.2023.1161396

**Published:** 2023-08-21

**Authors:** Rui Pan, Tian Hua, Qixin Guo, Hao Bai, Yong Jiang, Zhixiu Wang, Yulin Bi, Guohong Chen, Xinsheng Wu, Guobin Chang

**Affiliations:** ^1^ College of Animal Science and Technology, Yangzhou University, Yangzhou, China; ^2^ Joint International Research Laboratory of Agriculture and Agri-Product Safety, The Ministry of Education of China, Yangzhou University, Yangzhou, China

**Keywords:** duck, genomic selection signatures, *MITF*, beak color, SNP

## Abstract

**Introduction:** Beak color—a pigment-related trait—is an important feature of duck breeds. Recently, little research has addressed genetic mechanism of the beak colors in poultry, whereas the process and the regulation factors of melanin deposition have been well described.

**Methods:** To investigate the genetic mechanism of beak colors, we conducted an integrated analysis of genomic selection signatures to identify a candidate site associated with beak color. For this, we used black-billed (Yiyang I meat duck synthetic line H1, H2, H3&HF) and yellow-billed ducks (Cherry Valley ducks and white feather Putian black duck). Quantitative real-time PCR and genotyping approaches were used to verify the function of the candidate site.

**Results:** We identified 3,895 windows containing 509 genes. After GO and KEGG enrichment analysis, nine genes were selected. Ultimately, *MITF* was selected by comparing the genomic differentiation (F_ST_). After loci information selection, 41 extreme significantly different loci were selected, which are all located in intron regions of *MITF* and are in almost complete linkage disequilibrium. Subsequently, the site ASM874695v1:10:g.17814522T > A in *MITF* was selected as the marker site. Furthermore, we found that MITF expression is significantly higher in black-beaked ducks than in yellow-beaked ducks of the F_2_ generation (*p* < 0.01). After genotyping, most yellow-billed individuals are found with homozygous variant; at the same time, there are no birds with homozygous variant in black-billed populations, while the birds with homozygous and heterozygous variant share the same proportion.

**Conclusion:** *MITF* plays a very critical role in the melanogenesis and melanin deposition of duck beaks, which can effectively affect the beak color. The *MITF* site, ASM874695v1:10:g.17814522T > A could be selected as a marker site for the duck beak color phenotype.

## Introduction

There is some skin pigmentation in most animals, and this is often associated with age and the environment. In poultry, pigment-related traits are important features for numerous breeds (Jialang, 2016; Rui, 2019). The colors and patterns of feathers, furs, and skin are considered special and unique traits for many genetic populations. The beak colors of ducks can be divided into yellow, black, and heather, and these beak colors are mainly affected by eumelanin synthesis and deposition.

During the development of black beaks, melanin is synthesized and deposited in the melanosome through complex biochemical processes, including melanocyte development and migration, melanin synthesis and regulation, and melanosome migration (Lin and Fisher, 2007; Dorshorst et al., 2010; Bastonini et al., 2016). Duck melanogenesis could be driven by the expression of the *TYR*, *TYRP1*, and *DCT*, which *MITF* may induce under the regulation of several pathways such as Wnt signaling pathway (Iozumi et al., 1993; Lu et al., 2007; An et al., 2009; Lo and Fisher, 2014; Paterson et al., 2015; Allouche et al., 2021). Before the melanogenesis, EDNRB pathway plays an important role in the development of the melanocytes (Van Raamsdonk et al., 2004; Li et al., 2019; Wang et al., 2021). Subsequently, melanosomes migrate from the perinuclear region of melanocytes toward the plasma membrane in the presence of microtubules, actin filaments, and myosin, which ultimately affect the surrounding skin, hair, feathers, etc. (Hirokawa and Noda, 2008; Cieslak et al., 2011; Mica et al., 2013). Recently, little research has addressed genetic mechanism of the beak colors in poultry, whereas the process and the regulation factors of melanin deposition have been well described. In poultry, beak color is thought to be regulated by two pairs of autosomal alleles (C/c and Mb/mb; c has a recessive epistatic effect on mb) (Jialang, 2016). Assisted by recent rapid developments in genetics and genomics, several single-nucleotide polymorphisms in *MITF*, *EDNRB2*, *ASIP*, *MC1R*, and *POU2F3* have been significantly associated with the regulation of melanin synthesis and beak color of ducks (Guo et al., 2022; Liu et al., 2022).

In this study, Yiyang I meat duck synthetic line (H1, H2, H3&HF) was selected as black-billed ducks, Cherry Valley duck and white feather Putian Black Duck (Line E) were selected as yellow-billed duck for genomic selection signatures. The integrated analysis of F_ST_ and θπ was performed to select key genes and differential loci to regulate melanin deposition and formation of beak color in ducks. The candidate alleles and SNPs were selected for further validation. To detect the expression level in ducks with different beak colors, the bill tissues of each of five individuals randomly selected from a hybrid F_2_-generation population of Cherry Valley ducks and White Crested ducks, which is an indigenous breed with white feather and black beak, with different beak color (hereinafter referred to as F2 yellow and F2 black) was collected to perform real-time qPCR. Subsequently, the Cherry Valley duck and four other duck breeds (Jingding, Ji’an red, Gaoyou, and Liancheng White), along with F_2_ yellow and F_2_ black, were randomly selected for venous blood sampling. The PCR amplification method was used to verify the candidate SNPs. For this, PCR analyses were done for 30 birds from each group.

This paper provides a candidate genetic marker which can be applied in duck breeding for beak color. In addition, this research can provide a theoretical basis for the research of melanin, pigment-related traits in poultry and preservation of the crested white ducks.

## Materials and methods

### Ethical approval

All experiments on ducks were performed in accordance with the Regulations on the Administration of Experimental Animals issued by the Ministry of Science and Technology (Beijing, China) in 1988 (last modified in 2001). The experimental protocols were approved by the Animal Care and Use Committee of the Yangzhou University (YZUDWSY 2017-11-07). All efforts were made to minimize animal discomfort and suffering.

### Establishment of resource groups and sample collection

In this experiment, Yiyang I meat duck synthetic line (H1, H2, H3&HF) was selected as black-billed duck, Cherry Valley duck and white feather Putian black duck (Line E) selected as yellow-billed duck for the experiment. Among them, Yiyang I meat duck is a synthetic line with moderate growth rate, high feed conversion rate, strong stress resistance and good meat quality obtained from Shuyang Zhongke Breeding Co., Ltd. and Yangzhou University. First, the crossbreeding scheme are Beijing duck × Liancheng white duck, Liancheng white duck × Runzhou white crested duck and Beijing duck × Runzhou white crested duck. Then, individuals with black beak, white feathers and blue feet were selected to form H1, H2, H3&HF specialized strains which are bred for different traits, such as body weight. For whole-genome resequencing, ten birds of each variety or strain were selected for vein blood collection, and the blood samples were stored at −20°C. Thus, the F_2_ resource population, the result of crossing the Chinese Crested duck and Cherry Valley duck, was obtained from the Laboratory of Poultry Genetic Resources Evaluation and Germplasm Utilization at Yangzhou University.

The Runzhou white-crested duck used in this experiment came from Zhenjiang Tiancheng Agriculture Co., Ltd., and all other varieties or strains came from Shuyang Zhongke Breeding Poultry Co., Ltd. All the breeding experiments were completed at a breeding farm with scientific feeding and management to ensure clean and sufficient drinking water and sufficient nutrients. Single cage feeding was adopted to ensure the cleanliness and hygiene of the duck houses and to avoid the occurrence of infectious diseases. At 42 days old, the beak color separation ratio of 308 ducks from the F_2_ hybrid population was counted and analyzed. Colorimetric card alignment was used, and the melanin coverage was classified. Full coverage of melanin and black alignment were considered pure black, while zero melanin coverage and yellow alignment were defined as pure yellow. Next, in the F_2_ hybrid population, five black and yellow ducks were randomly selected for beak collection and were stored at −80°C. Later, the Cherry Valley duck and four indigenous breeds (Jingding, Ji’an red, Gaoyou, and Liancheng White), along with F_2_ yellow and F_2_ black, were randomly selected for venous blood collection (from 30 birds for each group).

### Whole-genome resequencing and SNP calling

The blood samples were selected for DNA extraction using the phenol–chloroform protocol and the quality was detected by the Nanodrop ND-2000 spectrophotometer and observing DNA sample appearance. Two-end libraries of the evaluated samples were built according to the Illumina Hiseq PE150 platform. Small fragment libraries with a fragment length of 150 bp were constructed, and the successfully constructed libraries were sequenced via the Illumina Hiseq PE150 platform.

For quality assessment and detection of clean reads acquired from sequencing, low quality and adaptor sequences were filtered out by trinmomtic. The filtered high-qualified sequences were aligned to the mallard genome (https://asia.ensembl.org/Anas_platyrhynchos/Info/Index?db=core) by BWA-mem, with the default alignment parameter for BWA-mem. The contrasted bam files were sorted using SAMtools and duplicate aligned sequences were removed using Picard. Sequences near indel were realigned using RealignerTargetCreator and IndelRealigner in GATK, while SNPs and indels within the genome were searched by Unified Genotyprer in GATK, and the SNPs and indels were filtered under: 1) QUAL > 30; 2) 18X > DP > 5X; 3) deletion rate < 0.1; 4) retained bialleles.

### Genomic selection signatures and principal components analysis

Integrated analysis of F_ST_ and θπ was performed to detect regions with significant signatures of selective sweep. In this experiment, a sliding-window approach (10-kb windows sliding in 5 kb steps) was applied to quantify polymorphism levels (θπ, pairwise nucleotide variation as a measure of variability) and genetic differentiation (F_ST_) between black- and yellow-beaked ducks. To analyze the distribution of the θπ ratios and F_ST_ values for Integrated analysis, we calculate log_2_ (θπ, black beak/θπ, yellow beak). Windows with significant extreme values (the 5% left and right tails) and significant high F_ST_ values (the 5% right tail) were selected as regions with strong selective sweep signals along the genome.

Principal component analysis (PCA) was performed using free software–GCTA (Yang et al., 2011), before which the database was created with SNP information of selected windows. The VCF file was transformed the format with software vcftools and plink.

All results above were visualized by R.

### Gene annotation and enrichment

The filtered windows were annotated using the VEP tool (https://asia.ensembl.org/Tools/VEP) and the reference genome is the mallard genome ASM874695v1 (https://asia.ensembl.org/Anas_platyrhynchos/Info/Index? The db=core) to o-btain the corresponding gene name and variant information. Corresponding gene-s were analyzed by GO and KEGG using the KOBAS platform (http://bioinfo.org/kobas/) and visualized by R after the calculated *p*-value were gone through FDR Correction, taking FDR ≤ 0.05 as a threshold.

### PCR amplification

The PCR amplification reaction mixture consisted of 2 × Taq enzyme Mix (12.5 µL), forward primer (1 µL), reverse primer (1 µL), genomic DNA template (50 ng), and ddH_2_O (up to 25 µL). The PCR primer information is shown in Table 1.

**TABLE 1:** Information of primer sequences.

Primer	Sequence (5′-3′)	Role
MI-F1	TCC​CCA​TTC​TCC​AAA​GGT​TAG​C	SNP analysis
MI-R1	TCC​CCG​TTG​TCC​CCA​CTT​ATT​A	SNP analysis
MITF-F	CAG​GAC​TAA​ACA​TCA​GCA​ACT​CGT​G	RT-qPCR
MITF-R	CTT​GGG​TAT​CAA​AGT​GCC​TAG​TTC​T	RT-qPCR
GAPDH-F	GGT​TGT​CTC​CTG​CGA​CTT​CA	RT-qPCR
GAPDH-R	TCC​TTG​GAT​GCC​ATG​TGG​AC	RT-qPCR

The PCR amplification reaction conditions were as follows: 95°C pre-denaturation for 5 min, 94°C denaturation for 30 s, 60°C annealing for 30 s, 72°C (60 s/kb), extension for 30 s, 35 cycles; final extension at 72°C for 10 min. The PCR products were detected by agarose gel electrophoresis and observed and analyzed using a Gel Imaging System (UVP, LLC, United States). Forward and backward primers (10 µL) and the PCR amplification products were purified and directly sequenced in Beijing by Tianyi Huiyuan Biotechnology Co., Ltd. and Liuhe BGI Technology Co., Ltd.

### Construction of *MITF* tissue expression profiles

After RNA extraction from tissue samples using conventional methods, the concentration of RNA was detected at a Nanodrop ND-2000 spectrophotometer. The first-strand cDNA reaction was configured using the TaKaRa kit: 2 × RT Mix (10 µL), HiScript W Enzyme Mix (2 µL), Oligo (dT) 23VN (1 μL), Random hexamers (1.0 µL), total RNA (1 µg) and RNase Free ddH_2_O (up to 20 µL). In the PCR instrument, the reaction conditions are set as follows: 25°C for 5 min, 50°C for 15 min, and 85°C for 2 min.

The cDNA samples were quantified using the T5 Fast kit (Nanjing Tsingke Biotechnology Co., Ltd.). The qPCR reaction mixtures were as follows: 2 × T5 Fast qPCR Mix (10 µL), 10 µM Primer F (0.8 µL), 10 µM Primer R (0.8 µL), 50× R OX Reference Dye II (0.4 µL), Template DNA (10 ng), and ddH_2_O (up to 20 µL). The reaction procedure was as follows: 95°C for 1 min, 95°C for 10 s, 60°C for 5 s, for a total of 40 cycles. Melt curve analysis (60°C ∼ 95°C + 1°C/cycle, holding time 4 s), and carried out centrifugation on PCR plate centrifuge at 4°C 6,000 rpm for 30 s. The primer information used is shown in Table 1. Gene expression levels were calculated using the 2^−ΔΔCT^ method.

### Statistical analysis

The test statistics were initially collected by Excel, and were analyzed by SPSS 22.0 software (Malik et al., 2020) to test the homogeneity of variance. After the results meet the analysis requirements, genotyping was analyzed by Chi-square test and the fluorescence results were analyzed by One-Way ANOVA, Duncan’s method used for multiple comparisons. lt was defined significant as *p* < 0.05. Figures was drew using the GraphPad Prism 8.0 software (Mitteer et al., 2018).

### LD analysis

The information of all selected variants was generated and calculated from genotyping data of 60 birds, 20 F_2_ yellow ducks along with 20 F_2_ black ducks by Excel and the LD analysis between extreme different SNPs was performed by using Haploview 4.2 (Kim et al., 2013).

## Results and analysis

### Genomic selection signatures of beak color

**FIGURE 1:**
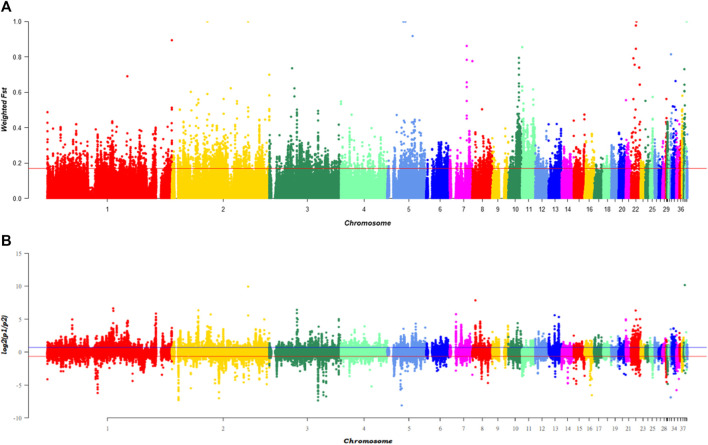
Genomic selection signatures of ducks with different beak colors, Note: **(A)** presents the distribution of weighed F_ST_ values in 60birds according to the genetic distance of windows, while **(B)** is the distribution of log2 [θπ(pop1/pop2)]. The red line represents the top 5% level of each index.

To identify candidate differentiation regions leading to black beak occurrence and serving as concomitant evolution during evolution, the genomic selection signatures was analyzed using 10 kb windows in black beak group (H1, H2, H3&HF) and yellow beak group (cherry Valley duck and Line E) with 5 kb steps. After sorting, the F_ST_ value of the top 5% of the window is above 0.17. Calculating log_2_ [θπ (pop1/pop2)] has found that the top 5% can reach 0.6545 and −0.6648 (Figures 1A, B). After screening of the intersection, a total of 3,895 windows were obtained, which contain 509 genes. In addition, PCA analysis was performed (Supplementary Figure S2) according to SNP loci of 3,895 windows on 60 birds, which contains 685,650 variants. As the result, black-beaked and yellow-beaked birds could be easily separated based on PC1. This result indicates that the SNP loci on selected windows are probably associated with beak colors.

For the GO and KEGG enrichment analysis of the 509 genes, and after correcting the *p*-value, we screened the melanin-related genes *MITF* and *SPARC* (Table 2). In addition, the significantly enriched KEGG pathways were shown in the bubble plot (Supplementary Figure S1) and the pathway related to melanin were selected (Table 3), which were Melanogenesis, MAPK signaling pathways and Wnt signaling pathway, including the genes, *ADCY1*, *ADCY2*, *ADCY5*, *DVL1*, *MITF*, *WNT7A*, *GNAI3*, *WNT11, ASIP* and so on.

**TABLE 2:** Information about the melanin-related GO terms.

ID	#Term	Adjusted *p*-value	Genes
GO:0030318	melanocyte differentiation	0.2262	*MITF*
GO:0043473	pigmentation	0.2413	*SPARC*

**TABLE 3:** Information about the melanin-related KEGG terms.

ID	#Term	Adjusted *p*-value	Genes
apla04916	Melanogenesis	0.0063	*ADCY5*, *ADCY1*, *ADCY2*, *DVL1*, *MITF*, *WNT7A*, *GNAI3*, *WNT11*, *ASIP*
apla04010	MAPK signaling pathway	0.0935	*RASGRP3*, *TGFB3*, *TRAF6*, *MECOM*, *CACNA1C*, *CACNA1G*, *STK3*, *MAPK11*, *MAPK12*, *ANGPT1*, *DUSP8*
apla04310	Wnt signaling pathway	0.2147	*RSPO2*, *PRICKLE2*, *RSPO1*, *DVL1*, *WNT7A*, *WNT11*
apla00350	Tyrosine metabolism	0.2896	*DDC*, *ALDH1A3*

### *MITF* locus screening and LD analysis

According to the standard of FDR < 0.05, only the term melanogenesis was selected for further validation. Furthermore, all the other genes have been confirmed to regulate *MITF* expression through the Wnt signaling pathway and cAMP-dependent signaling pathway (Gruis and van Doorn, 2012; Kim et al., 2018; Pruller et al., 2022). Thus, *MITF* is the most likely candidate gene for melanin disposition and formation of the black beak in ducks.

**FIGURE 2:**
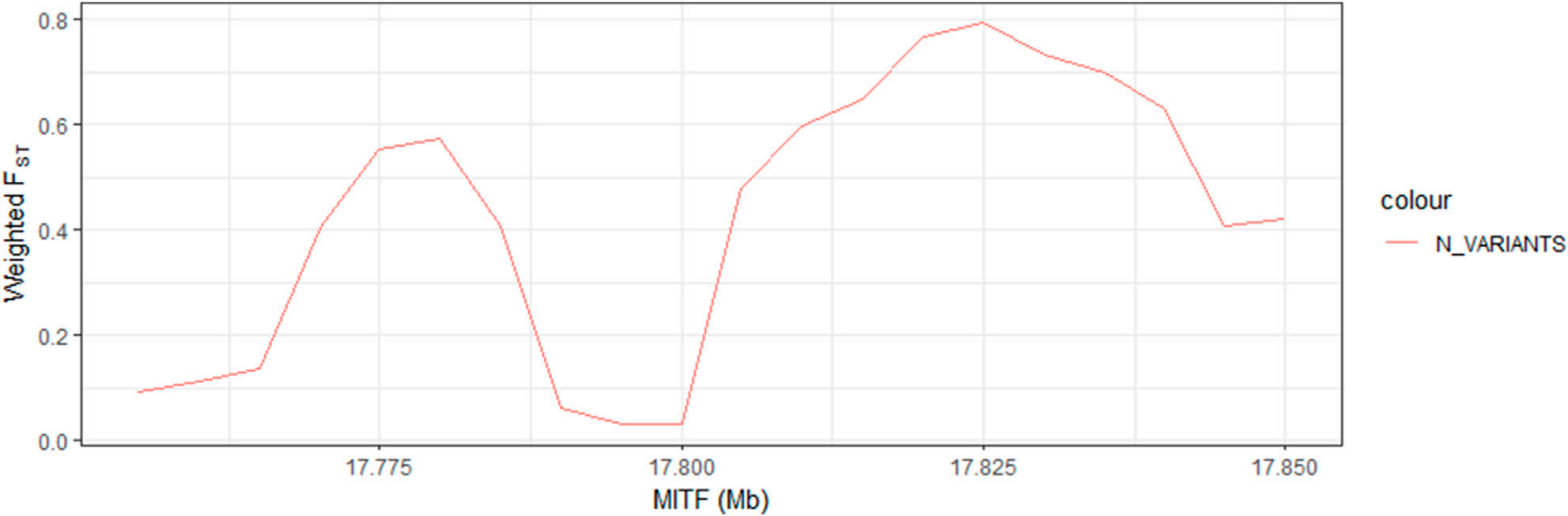
Selective clearing map of *MITF* based on weighted F_ST_.

To acquire more evidence for the inference above, we compare the distribution of the F_ST_ values for nine selected genes (Figure 2; Supplementary Figure S3). *MITF* had the highest F_ST_ values. In addition, information on all variants was collected, and the frequency of different types was calculated. Ultimately, we selected 41 variants, which is an extreme difference between birds with different beak colors (Table 4). It is worth mentioning that they are all in the intron regions of *MITF*. Furthermore, LD analysis of the extreme significant difference loci in *MITF* revealed that most of the loci were highly linked (Supplementary Figure S4). Therefore, MITF can be used as a candidate gene for forming black beaks.

**TABLE 4:** Information about the 41 extreme different loci.

#CHROM	POS	REF	ALT		Yellow beak (birds and frequency)			Black beak (birds and frequency)		
					Ref/ref	Ref/alt	Alt/alt	Ref/ref	Ref/alt	Alt/alt
10	17813533	G	T	intron_variant	0	0	20 (1)	40 (1)	0	0
10	17813579	C	G	intron_variant	0	0	20 (1)	40 (1)	0	0
10	17813659	C	T	intron_variant	0	0	20 (1)	40 (1)	0	0
10	17813681	G	A	intron_variant	0	0	20 (1)	40 (1)	0	0
10	17813845	C	T	intron_variant	0	0	20 (1)	40 (1)	0	0
10	17813883	C	T	intron_variant	0	0	20 (1)	39 (0.975)	1 (0.025)	0
10	17813989	T	C	intron_variant	0	0	20 (1)	40 (1)	0	0
10	17814232	C	T	intron_variant	0	0	20 (1)	40 (1)	0	0
10	17814486	C	A	intron_variant	20 (1)	0	0	0	1 (0.025)	39 (0.975)
10	17814522	T	A	intron_variant	0	0	20 (1)	40 (1)	0	0
10	17816174	T	C	intron_variant	0	0	20 (1)	40 (1)	0	0
10	17816775	T	C	intron_variant	0	0	20 (1)	40 (1)	0	0
10	17816914	A	G	intron_variant	20 (1)	0	0	0	0	40 (1)
10	17817511	C	T	intron_variant	0	0	20 (1)	40 (1)	0	0
10	17817645	G	A	intron_variant	0	0	20 (1)	40 (1)	0	0
10	17819421	G	A	intron_variant	0	0	20 (1)	40 (1)	0	0
10	17819741	T	C	intron_variant	20 (1)	0	0	0	0	40 (1)
10	17821300	A	G	intron_variant	0	0	20 (1)	40 (1)	0	0
10	17821305	T	G	intron_variant	0	0	20 (1)	40 (1)	0	0
10	17821868	C	T	intron_variant	0	0	20 (1)	40 (1)	0	0
10	17821942	T	A	intron_variant	0	0	20 (1)	40 (1)	0	0
10	17822058	G	A	intron_variant	0	0	20 (1)	40 (1)	0	0
10	17822348	A	G	intron_variant	0	0	20 (1)	40 (1)	0	0
10	17822368	G	A	intron_variant	0	0	20 (1)	40 (1)	0	0
10	17822496	G	A	intron_variant	0	0	20 (1)	40 (1)	0	0
10	17822600	T	G	intron_variant	0	0	20 (1)	40 (1)	0	0
10	17822617	G	A	intron_variant	0	0	20 (1)	40 (1)	0	0
10	17822689	G	T	intron_variant	0	0	20 (1)	40 (1)	0	0
10	17822972	C	G	intron_variant	0	0	20 (1)	40 (1)	0	0
10	17822994	G	A	intron_variant	0	0	20 (1)	40 (1)	0	0
10	17823136	A	G	intron_variant	0	0	20 (1)	40 (1)	0	0
10	17823139	C	T	intron_variant	0	0	20 (1)	40 (1)	0	0
10	17823565	C	T	intron_variant	0	0	20 (1)	40 (1)	0	0
10	17823728	G	A	intron_variant	0	0	20 (1)	40 (1)	0	0
10	17823907	C	T	intron_variant	0	0	20 (1)	40 (1)	0	0
10	17824027	A	G	intron_variant	0	0	20 (1)	39 (0.975)	1 (0.025)	0
10	17824405	T	C	intron_variant	0	0	20 (1)	40 (1)	0	0
10	17825466	G	C	intron_variant	0	0	20 (1)	40 (1)	0	0
10	17826419	G	A	intron_variant	0	0	20 (1)	40 (1)	0	0
10	17826502	A	G	intron_variant	20 (1)	0	0	0	1 (0.025)	39 (0.975)
10	17827159	C	T	intron_variant	0	1 (0.05)	19 (0.95)	40 (1)	0	0

Note: The number of POS, represents the position on chromosome 10 in genome ASM874695v1, and the selected site is marked in red in table.

### Analysis of *MITF* gene expression differences among individuals with different beak colors

**FIGURE 3:**
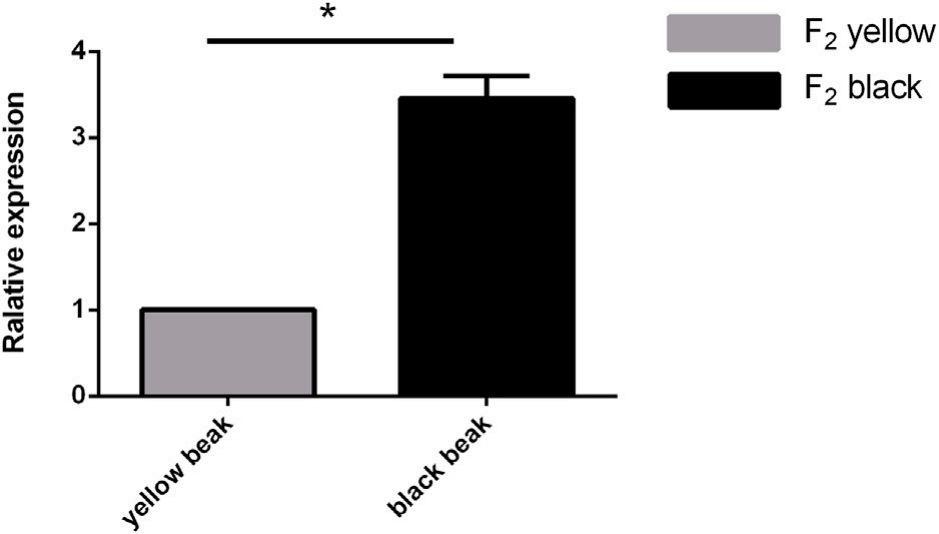
*MITF* expression levels in beak tissues of F_2_ yellow ducks and F_2_ black ducks.

The caracal tissues of individuals with different beak colors were selected to detect the expression levels of *MITF* using quantitative real-time PCR (Figure 3). The results show that the expression of *MITF* in the black duck beak tissue was significantly higher (*p* < 0.01) than that in the yellow beak group, further verifying that *MITF* is highly associated with beak colors.

### Validation of the marker site in *MITF*

In order to validate the association between 41 extreme different loci in *MITF* and beak color, genotyping of 41 loci should be performed. As the 41 loci were highly linked, only one locus, which is ASM874695v1:10: g.17814522T>A, was genotyped for validation. (The information of the variants used for the validation is shown in Table 5).

**TABLE 5:** Information about the selected site in *MITF*.

Chromosome	Position	Ref	Alt	Beak color (numbers)					
				Yellow			Black		
10	17814522	T	A	TT	AT	AA	TT	AT	AA
				0	0	20	40	0	0

The validation results of the marker SNP in *MITF* for 20 F_2_ yellow and F_2_ black individuals are shown in Table 6. In yellow-billed birds, there are no individuals with the genotype of TT, while proportion of homozygous individuals with the genotype of AA reaches 90%. While in birds with black beak, no homozygous variants were found, and the frequency of the birds with the genotype of TT was as the same as that of birds with the genotype of TA. Frequency statistics of the candidate SNP of *MITF* show that the probability of the variants A in the yellow-beaked birds is 95%, compared to 25% in the black-beaked individuals. Chi-square test for gene frequencies using SPSS software showed a level of significantly different (χ^2^ = 40.833, *p* < 0.001).

**TABLE 6:** Genotyping results of the selected sites in *MITF* with birds in the hybrid F_2_ population.

MITF: T-17814522-A	Genotype numbers (frequency)			Gene frequency	
	TT	TA	AA	T	A
F_2_ yellow	0	2 (10%)	18 (90%)	0.05	0.95
F_2_ black	10 (50%)	10 (50%)	0	0.75	0.25
χ^2^ = 40.833, *p* < 0.001					

Note: The locus number represents the position on chromosome 10 in genome ASM874695v1.

In addition, among the Cherry Valley, Jinding, and Ji’an Red ducks, the frequency of birds with a homozygous genotype with variants of AA were significantly different from that of the F_2_ black (*p* < 0.001). The χ^2^ values resulting from the chi-square tests were 68.157, 51.321, and 51.321, respectively (Table 7). On the contrary, no homozygous variants were found in the Gaoyou and Liancheng White ducks. Among them, birds with no variants account for more than 80% of Gaoyou ducks, while more than half of individuals in Liancheng White ducks have no variants, significantly different from yellow-billed individuals (*p* < 0.001). The chi-square test generated χ^2^ values of 49.932 and 31.689, respectively (Table 8). Based on these findings, the candidate SNP of *MITF* was significantly associated with the beak color.

**TABLE 7:** Results of genotyping and Chi-square test for the selected site in *MITF* in yellow-beaked duck.

Variety	Genotype numbers			Gene frequency		Chi-square value	*p*-value
	TT	TA	AA	T	A		
Cherry Valley Duck	0	0	30	0	1	68.2	<0.0001
Jingding Duck	0	0	20	0	1	51.3	<0.0001
Ji’an Red Duck	0	0	20	0	1	51.3	<0.0001

Note: All Chi-square test is performed between corresponding breed and F_2_ black individuals.

**TABLE 8:** Results of genotyping and Chi-square test for the selected site in *MITF* in black-beaked duck.

Variety	Genotype numbers			Gene frequency		Chi-square value	*p*-value
	TT	TA	AA	T	A		
Gaoyou Duck	24	5	0	0.91	0.09	49.9	<0.0001
Liancheng White Duck	17	13	0	0.78	0.22	31.7	<0.0001

Note: All Chi-square test is performed between corresponding breed and F_2_ yellow individuals.

## Discussion

To identify candidate alleles and SNPs that lead to melanin deposition and formation of black beak, population selection signature was performed by integrated analysis of F_ST_ and θπ using 10 kb windows to ensure that a single window contained only 1–2 alleles. In the experiment, F_ST_ and θπ were selected for joint analysis, where F_ST_ was based on population differentiation, detecting the potential selection region according to the polymorphism of the SNP loci (Lewontin and Krakauer, 1973; Yunlong, 2015), and the θπ is defined as nucleotide polymorphism. When a specific region on the genome is subject to selection, the region and its linked regions exhibit decreased polymorphism along with increased purity and degree due to the presence of “selective clearance” (Nei and Li, 1979; Kilian et al., 2007; Shapiro et al., 2013). As a result, after analysis under 10-kb windows between two groups divided from six populations with further genetic distance, there are multiple windows with F_ST_ = 1. Therefore, there are multiple regions that make significant contributions to beak color. And it should be feasible to obtain stable black beaks by breed according to these regions.

Comprehensive analysis of the two indicators revealed that the 509 genes were strongly selected, in which *ADCY1*, *ADCY2*, *ADCY5*, *DVL1*, *MITF*, *WNT7A*, *GNAI3*, *WNT11*, *ASIP* were closely related to melanogenesis, which might closely relate to beak colors in duck. As the main regulator of melanin, *MITF* can regulate the transcription of *TYR*, *TRP-1* and *TRP-2* by binding the promoter factor region (Otreba et al., 2012; Wang et al., 2014; Jia et al., 2021). Moreover, as the key genes on the WNT signaling pathway and CAMP signaling pathway (Kim et al., 2018; Chen et al., 2020; Pallotti et al., 2020; Dommel et al., 2021; Pruller et al., 2022) all the other selected genes have been confirmed to regulate *MITF* expression (Khaled et al., 2002; Huber et al., 2003; Otreba et al., 2012; Lee et al., 2016). Furthermore, *MITF* have been found to play key regulatory roles in multiple pigment-related traits. Mochii et al. (1998) studied the reason of neuroretina abnormalities in Japanese quail and it was found that the *MITF* variants showed silver and white feathers and also showed neuroretina abnormalities. In addition, The *MITF* isoform MITF-M is highly expressed in duck black hair balls, but is not seen in white hair balls, and the upregulation of MITF-M was suggested to promote the formation of black feathers (Lin et al., 2019). Recently, it was also reported that *MITF* is associated with the duck-recessive white feather locus C, and that the 6.6 KB insertion on the *MITF* intron causes duck feather becoming white (Zhou et al., 2018). Taken together, these findings show that *MITF* plays a critical and direct role in the generation of melanin, which can effectively affect the beak color. It is worth mentioning that the selected genes are all associated with melanin synthesis, therefore, the differences between duck beak color are mainly derived from the differences in beak melanin synthesis.

Though *MITF* plays an important role in ducks’ beak color, the other genes’ functions cannot be ignored. Therefore, all nine selected genes were considered in the following selection. As a result, *MITF* holds the highest F_ST_ values and is much higher than the other genes. Therefore, compared with the other genes, *MITF* has a higher polymorphism level. Because of the limited sample size, only extremely different loci were selected as candidate sites, meaning that the sequence of the “ref” could be barely found in birds with one type of beak color while the sequence of the “alt” could be found in another only. According to the standard above, 41 loci in the intron regions of *MITF* were selected, while no extremely different loci were found in the other genes. In addition, the relative expression of *MITF* in black-beaked individuals was significantly higher than that in yellow-beaked individuals (*p* < 0.01). Therefore, *MITF* could be used as a candidate region affecting the beak colors of ducks and the 41 selected loci in *MITF* were considered candidate genetic markers of beak colors.

Finally, we select one of 41 extreme significant loci as the marker locus that regulates the melanogenesis and melanin deposition of duck beaks, given that the 41 extreme different loci are highly linked by the LD analysis. The locus was verified in five more populations, and the results showed that the three yellow-billed breeds (Cherry Valley, Jinding, and Ji’an Red) were all AA type, while 80% birds from the black-billed breeds (Gaoyou and Liancheng White ducks) were of TT type. Similar results were obtained in the hybrid F_2_ generation population. Thus, the locus could be used to completely distinguish the yellow-billed ducks from the black-billed ducks. Among the black-beaked individuals, both the TT type and TA type were found. It indicates that different beak colors could not be just explained by variants of *MITF*. Further experiments are acquired to study the genetic mechanism of beak colors in duck. It is worth mentioning that all ducks with the homozygous variants of AA were yellow-beaked. Therefore, this site can be used as a yellow-beak marker. When applied to production, ducks can be bred for beak color with AA type selection for the yellow beak phenotype.

## Conclusion

Our experiments confirm that *MITF* has a significant effect on beak color. The *MITF* site ASM874695v1:10:g.17814522T > A is significantly different in the yellow-beaked and black-beaked individuals and should be considered a marker site for duck beak color phenotype.

## Data Availability

The data presented in the study are deposited in the NCBI repository, accession number PRJNA1004892.

## Supplementary material

SUPPLEMENTARY FIGURE S1 The GO and KEGG enrichment of selected windows. Note: A is the bar plot representing the top 5 MF, CC and BP terms of GO enrichment, while B is the bubble plot representing the top 20 KEGG terms of KEGG enrichment.

SUPPLEMENTARY FIGURE S2 PCA analysis of 60 birds with different beak colors.

SUPPLEMENTARY FIGURE S3 Selective clearing map of selected genes based on weighted F_ST_.

SUPPLEMENTARY FIGURE S4 LD heatmap of extreme different loci of selected windows in *MITF*. Each locus in selected windows was performed the chi-square test by SPSS. All significant loci are visualized in LD heatmap which is depicted in red.
