# Supplementary figures and images for: Identification of SNPs in MITF associated with beak color of duck
Source: Front Genet. 2023 Aug 21;14:1161396. doi: 10.3389/fgene.2023.1161396 (PMC10475569; doi:10.3389/fgene.2023.1161396)

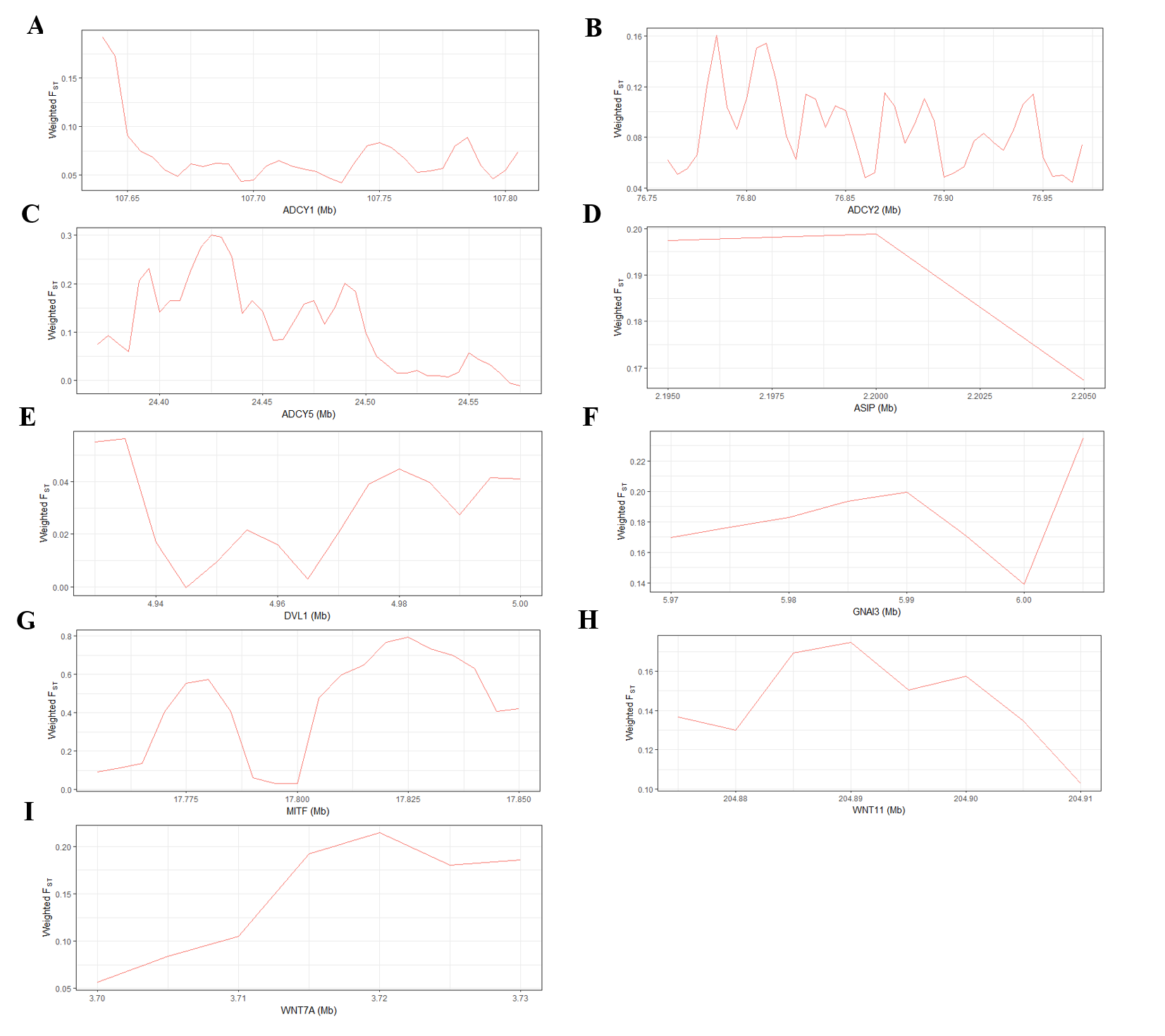

Supplement: Supplementary file 1 [file Image3.TIF]

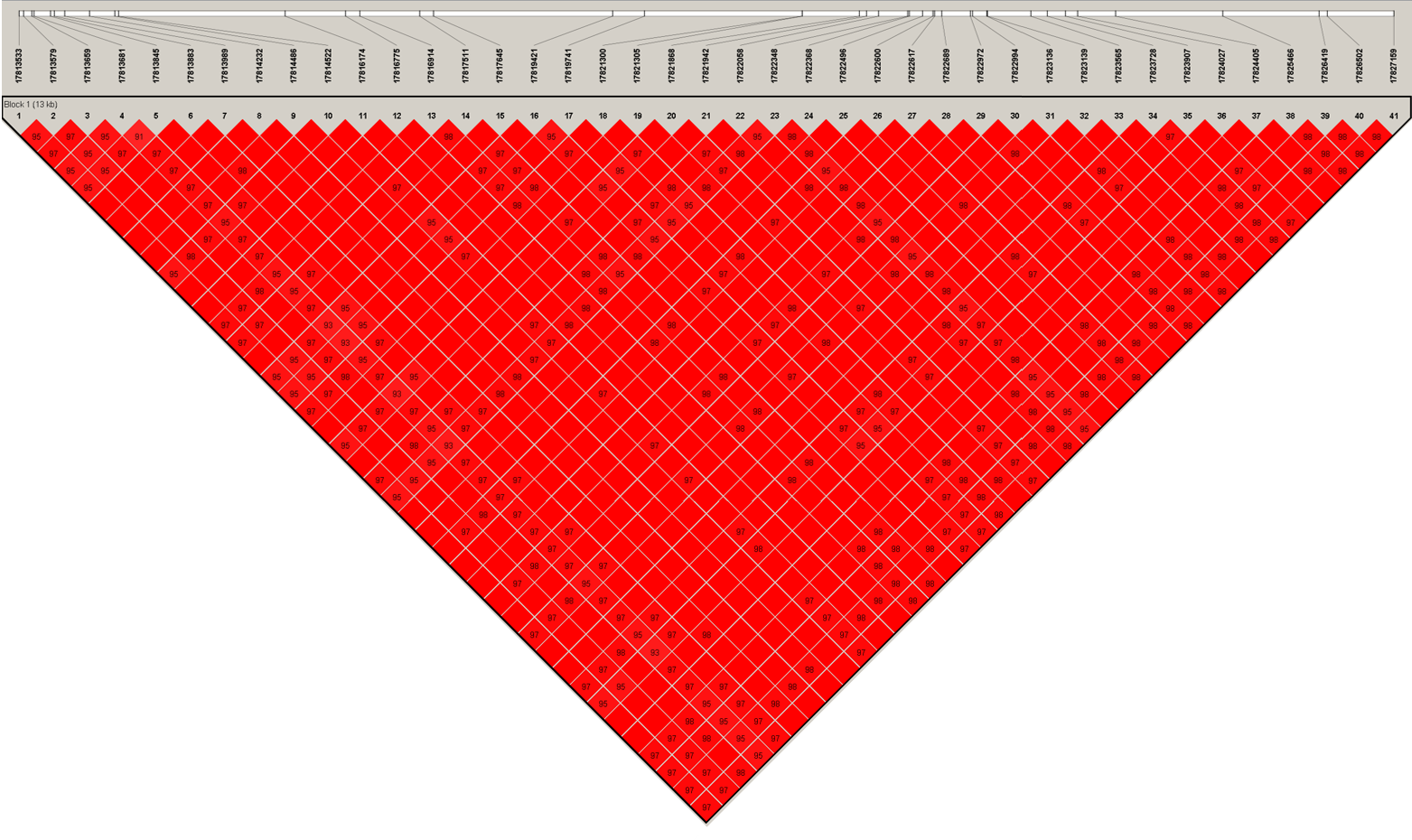

Supplement: Supplementary file 2 [file Image4.TIF]

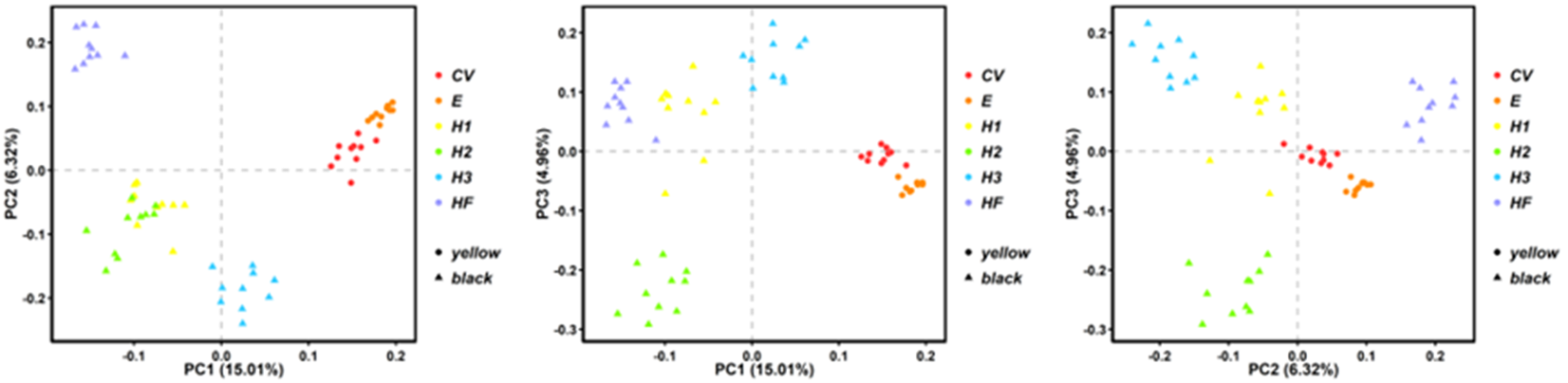

Supplement: Supplementary file 3 [file Image2.TIF]

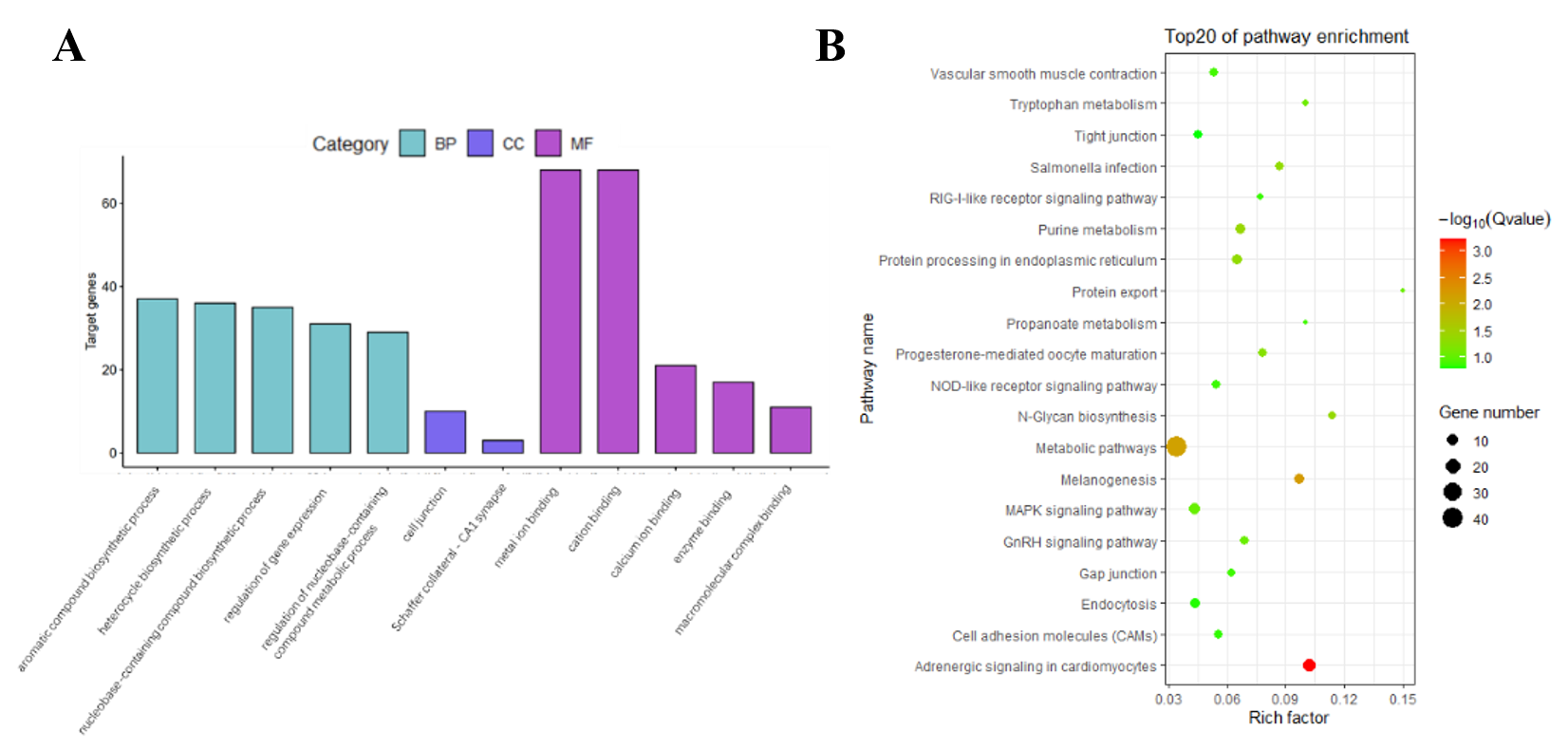

Supplement: Supplementary file 4 [file Image1.TIF]
